# Pulp sensitivity changes during orthodontic treatment at different time periods: **a** prospective study

**DOI:** 10.1007/s00784-020-03651-4

**Published:** 2020-12-07

**Authors:** Benjamín Briseño-Marroquín, Héctor López-Murillo, Robert Kuchen, Adán Casasa-Araujo, Thomas Gerhard Wolf

**Affiliations:** 1 Department of Restorative, Preventive and Paediatric Dentistry, School of Dental Medicine, University of Bern, Freiburgstrasse 7, CH-3010 Bern, Switzerland; 2 Department of Periodontology and Operative Dentistry, University Medical Center, Johannes Gutenberg University Mainz, Mainz, Germany; 3 Center for Advanced Studies in Orthodontics A.C., Mexico City, Mexico; 4 Institute for Medical Biometrics, Epidemiology and Informatics, University Medical Center, Johannes Gutenberg University Mainz, Mainz, Germany

**Keywords:** Electric pulp tester, Pulp sensitivity, Orthodontic treatment, Orthodontic forces

## Abstract

**Objective:**

The purpose of this investigation was to recognize pulp sensitivity changes in teeth receiving orthodontic treatment by means of an electric pulp tester (Vitality Scanner Model 2006; Kerr Corporation, Brea CA, USA).

**Materials and methods:**

An electric stimulus response threshold of eight teeth in 22 patients was measured prior to positioning orthodontic attachments, immediately before ligation of a nickel titanium archwire, immediately after ligation of a stainless steel archwire and 9 to 15 months after having achieved the clinical purposes established with the nickel titanium archwires. The first measurement served as baseline.

**Results:**

All teeth responded to an electrical stimulus at all times. No statistical differences were observed between the response thresholds obtained at different treatment times. The mean response threshold of the second measurement showed a decreasing response threshold tendency when compared with those of the baseline measurement. The mean response threshold of the third measurement showed an increasing tendency when compared with those of the baseline measurement. The first maxillary incisor and canine showed the lowest decreasing response threshold after the second measurement and the highest increasing response threshold after the third measurement. Less noticeable, but similar decreasing and increasing response threshold tendencies were observed in all other teeth after the second and third measurements, respectively.

**Conclusions:**

The results obtained in this investigation suggest that pulp sensitivity can be monitored during orthodontic treatment by means of an electric pulp tester.

**Clinical relevance:**

The importance of monitoring the pulp status during orthodontic treatment.

## Introduction

The dental profession is yet to establish a simple, objective, standardized, reproducible, noninvasive, and accurate method to diagnose diseases of the dental pulp. Electric pulp testing is a sensitivity pulp testing method based on stimulation of sensory nerves that relies on a given patient’s subjective reaction. Thus, false-positive and false-negative results should always be considered. Electric pulp testing, when properly utilized, is nevertheless safe and can provide clinical information regarding the pulp health status [1–3].

Magitot [4] was the first author to advocate the use of electricity in dentistry in 1878. Thirteen years later, Marshall [5] stated “As a means of diagnosis in obscure cases of the vitality or non-vitality of the dental pulp, I know of nothing so sure to demonstrate to a positive certainty these conditions as the electrical currents, both the galvanic and the faradic. In the more obscure cases, however, the faradic is superior to the galvanic, for if there is the slightest vitality remaining in the pulp, it will demonstrate it instantly by causing a response in the tooth.” In 1896, Woodward (cited by Prinz [6]) disclosed “If a few cells of a cataphoric electrode be applied to the dentin or metallic filling in a vital tooth, while the negative the pole is at the cheek or wrist of the patient, a distinct sensation should be felt, while in case of dead pulp there will be no response; usually even a small filling will transmit a distinct shock in a vital tooth, which is absent in a devitalized tooth. A mild interrupted current has also been used for the test.” In 1930, Ziskin and Wald [7] reported that “There appear to be favorable frequency ranges determining thresholds of stimulation in pulp testing.” In 1935, Kaletsky and Furedy [8] and subsequently Markus [9] in 1946 were among the first authors to investigate the effects of orthodontic forces on pulp vitality by means of an electric pulp tester. Markus [9] reported that “the threshold of stimulation was lowered, which is indicative of pulpal irritation.” He concluded that “it may be stated from the foregoing that proper and accurate pulp-testing, especially of the upper four incisor teeth, should be of value to the orthodontist to determine the status of the pulps prior to treatment, thus safeguarding the patient and the orthodontist.” In the 1960s, Seltzer et al. [3] and Lundy and Stanley [10] concluded that electric pulp testers were capable of detecting pulp changes only in cases when a major inflammatory response had occurred. They also reported that although no correlation between the electric pulp test reading and a specific histopathologic pulp condition could be established, a negative reading will occur in case of a necrotic pulp. The most common electric pulp testers are battery operated (monopolar). Monopolar and bipolar pulp testers are based on the production of impulses of negative polarity which reduce the voltage required to stimulate a nerve response in the pulp, without stimulating the periodontium nervous tissue [11]. Pulp testers produce different electric impulses which can be increased manually or automatically, depending on the device utilized [12, 13].

It has been reported that excessive orthodontic forces have an influence on the cementum hardness and elastic modulus [14] and can cause periodontal inflammation [15, 16]. Other research groups report an immediate pulp hypoxia effect, pulp tissue changes [17–19], and root resorption [20] after orthodontic forces are applied. Other investigations [21, 22] have reported that orthodontic treatment can cause a pulp sensitivity increase and metabolic changes expressed by increased activity of aspartate aminotransferase. Pulpal blood flow reduction [23], vacuolization, and disruption of the odontoblastic layer [24] have also been reported. Moreover, a calcitonin gene-related peptide (CGRP) expression increase in the pulp [25] due to excessive orthodontic forces and an increase in angiogenic growth factors in the pulp during orthodontic treatment [26–29] have been observed. Various research groups [30, 31] have also reported that pain caused during orthodontic treatment can be explained, to a certain extent, through the role that biologically active neuropeptides play in tissue inflammation and neuromodulation. Higher levels of inflammatory mediators have been likewise identified in pulp tissues and in gingival crevicular fluid during orthodontic treatment [16, 32–34]. However, other research groups [35, 36] reported no significant effect of orthodontic intrusive and/or extrusive forces on pulpal blood flow. In addition, Consolaro and Bianco Consolaro [37] claim that while light to moderate orthodontic forces applied to the periodontal ligament will promote periodontal cellular stress, which may evolve into a mild inflammation for some hours or days, they will recede in 2 to 7 days allowing periodontal reorganization between 10 and 15 days after the activation of orthodontic attachments.

In accordance with such evidence, it was expected in this investigation that the application of orthodontic forces would produce pulp tissue changes and periodontal inflammation. Thus, the aim of this investigation was to determine with an in vivo prospective investigation protocol if differences on pulp sensitivity status could be recognized by means of electric pulp testing in pre-selected teeth during long-term active orthodontic treatment.

## Materials and methods

### Patient/teeth selection and treatment protocol

A total of 39 patients, 14 to 48 years of age needing orthodontic treatment, were enrolled in this prospective study. Patient-written consent was obtained prior to enrollment. An orthodontic treatment plan was established after patient screening examination, extraoral facial assessment, intraoral examination, muscular and functional temporomandibular joint status evaluation, extra and intraoral images, dental casts, and intraoral and/or panoramic and cephalometric radiographs were completed and evaluated at the Center for Advanced Studies in Orthodontics in Mexico City. The treatment plan was periodically reassessed; thus, the short- and/or long-term objectives were accordingly modified. The diagnoses and orthodontic treatment procedures as well as devices and materials implemented in this investigation pertain to well-accepted clinical procedures; thus, it was established that only patient-written consent should be obtained prior to enrollment. A clinical trial registration was not required in the corresponding clinical facilities at the time of patient enrollment.

Eight teeth: the incisor, canine, premolar, and molar groups from each patient—11, 13, 15, and 16 (8, 6, 4, and 3 according to the American Dental Association Universal Numbering system, respectively) from the first quadrant and 31, 33, 35, and 36 (24, 22, 20, and 19 according to the American Dental Association Universal Numbering system, respectively) from the third quadrant—were selected for evaluation with an electric pulp tester (Vitality Scanner Model 2006; Kerr Corporation, Brea CA, USA). Exclusion criteria included previous orthodontic treatment, those scheduled for maxillofacial surgery, major systemic disease, medication, pregnancy, a periodontal probing depth higher than 3 mm, radiographic bone loss, endodontically treated teeth, history of trauma, and radiographically discernible, incomplete root development. Immediately after the oral diagnosis, the pre-selected teeth were isolated by means of a cotton roll; their surfaces air-dried; a conductive medium (toothpaste) was applied; and the probe tip of the electric pulp tester and the corresponding measurement was carried out. The buccal surface of each tooth was divided into six squares, and one of them was selected for the electric measurement in order to ensure the reproduction of the measurement and the information obtained [38]. The square selected for the baseline and further measurements was designated in a way that no contact between the electric pup tester-tip or the conductive medium and neighboring tissues and/or archwire could be possible, thus, avoiding an electric impulse conduction to the neighbor teeth. The panel wheel of the electric pulp tester was set at 3 at all times. The electric pulp tester was turned on automatically after establishing a stable contact between the probe and the tooth. The electrical intensity stimulus increased automatically. Patients were instructed to give a hand or voice signal after they had perceived the stimulus transmitted by the probe. The corresponding unit reading (from 0 to 80) was accordingly protocoled.

The first electric pulp testing was conducted after the oral diagnosis was completed and prior to the bonding of the orthodontic brackets. The results of the baseline measurement served as stimuli response threshold baseline for further measurements. The second electric stimuli response threshold was determined immediately after the brackets were bonded and having placed a 0.014-inch nickel titanium archwire (Stylus; Ahkimpech, S.A. de C.V., Mexico City, Mexico). The third electric stimuli response threshold was determined after having achieved the clinical purposes established with the nickel titanium archwires (between 9 and 15 months) immediately after having placed a 0.016-inch round stainless steel archwire (Stylus; Ahkimpech, S.A. de C.V., Mexico City, Mexico).

### Statistical analysis

The differences of the teeth groups individually, as well as all teeth groups together at the 1st–2nd, 1st–3rd, and 2nd–3rd measurement points, were statistically analyzed. The results were descriptively analyzed, and categorical variables are indicated with relative (%) frequencies. Continuous, discrete variables are indicated with arithmetic mean, and standard deviation (SD), median, and 25%/75% quantiles are depicted by box plots, minimum (Min), and maximum (Max). Normal distribution of continuous/discrete variables was assessed by histograms. The null hypothesis states that the mean pulp sensibility threshold changes between the three different measurement-point pairs are equal to 0. To account for the fact that eight teeth are nested within the same patient, a multilevel model is fitted. This model captures the patient-specific heterogeneities by including one random intercept for each measurement-point pair of the same patient (resulting in a total of 3 ∗ 22 = 66 estimated random intercepts). The main interest lies in the three fixed intercepts, which quantify the mean changes in the sensibility thresholds between the three measurement-point pairs. Three Wald tests check whether the estimates of these intercepts are significantly different from 0.

## Results

**Fig. 1:**
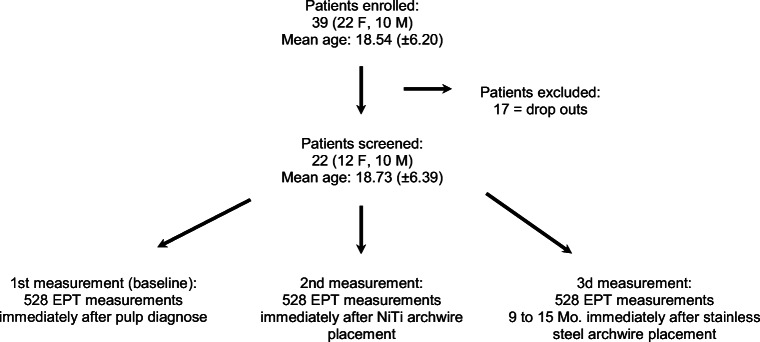
Flow diagram of collected data of all teeth when considering the clinical inclusion and exclusion parameters (EPT = electric pulp tester)

Twenty-two out of 39 enrolled patients (14 to 36 years of age ± 6.39; 12 females, 10 males) completed the orthodontic treatment. A total of 528 baseline and second and third electric pulp tester measurements were made (Fig. 1). The time span between the second and third measurements was between 9 and 15 months (Ø 12.7 months; ± 1.89). Table 1 summarizes the descriptive analysis of the data of the electric pulp tester response thresholds obtained in this investigation at the baseline and second measurements.

**Table 1:** Descriptive analysis of an electric pulp tester unit readings of pre-selected maxillary and mandibular teeth from 22 patients obtained during active orthodontic treatment (Meas. = measurements: 11–8, 13–6, 15–4, and 16–3 maxillary right; 31–24, 33–22, 35–20, and 36–19 mandibular left side according to the Federation Dentaire International and American Dental Association Universal Numbering systems, respectively)

	Age	Tooth 11–8/meas.			Tooth 13–6/meas.			Tooth 15–4/meas.			Tooth 16–3/meas.		
		1st	2nd	3rd	1st	2nd	3rd	1st	2nd	3rd	1st	2nd	3rd
Mean	18.76	28.86	29.36	30.27	32.09	32.09	33.86	34.36	33.59	34.09	35.05	33.86	34.27
SD	6.54	9.87	8.02	6.65	9.43	7	6.17	7.56	7.64	7.89	11.84	11.6	10.74
Min.	14	0	15	21	0	20	23	20	20	21	17	16	16
Max.	36	48	52	48	52	50	48	47	47	50	53	53	53
	Age	Tooth 31–24/meas.			Tooth 33–22/meas.			Tooth 35–20/meas.			Tooth 36–19/meas.		
		1st	2nd	3rd	1st	2nd	3rd	1st	2nd	3rd	1st	2nd	3rd
Mean	18.76	30.91	29.77	31.68	32.68	31.64	33.36	33.27	32.41	32.09	38.32	37.91	37.77
SD	6.54	10.06	10.33	8.27	7.17	6.9	6.79	9.19	8.13	9.36	9.55	9.09	9.03
Min.	14	15	12	20	21	22	24	14	16	14	25	26	24
Max.	36	50	49	48	49	48	45	46	47	46	56	53	53

**Fig. 2:**
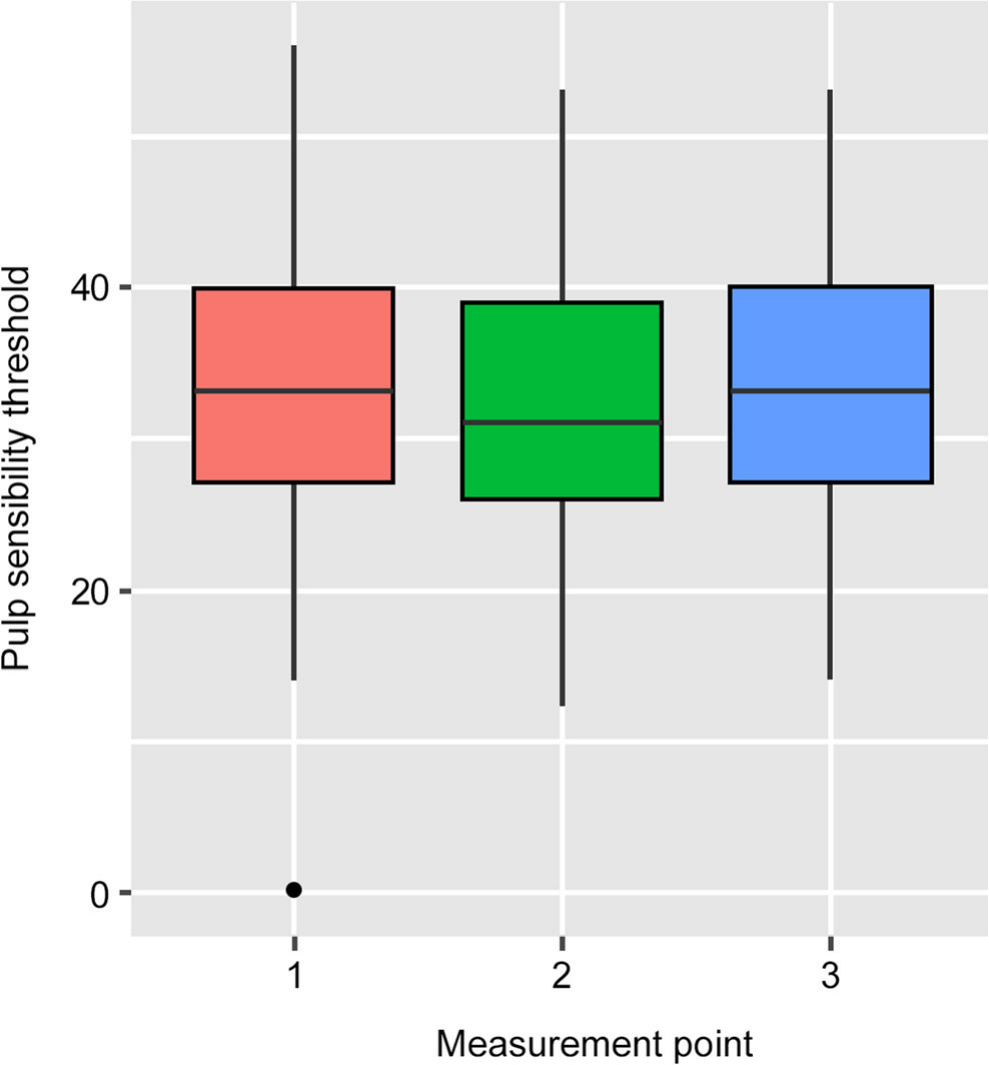
Box plots summarizing the absolute values of the pulp sensibility thresholds response at the different measurement points according to the pulp tester response scale

**Fig. 3:**
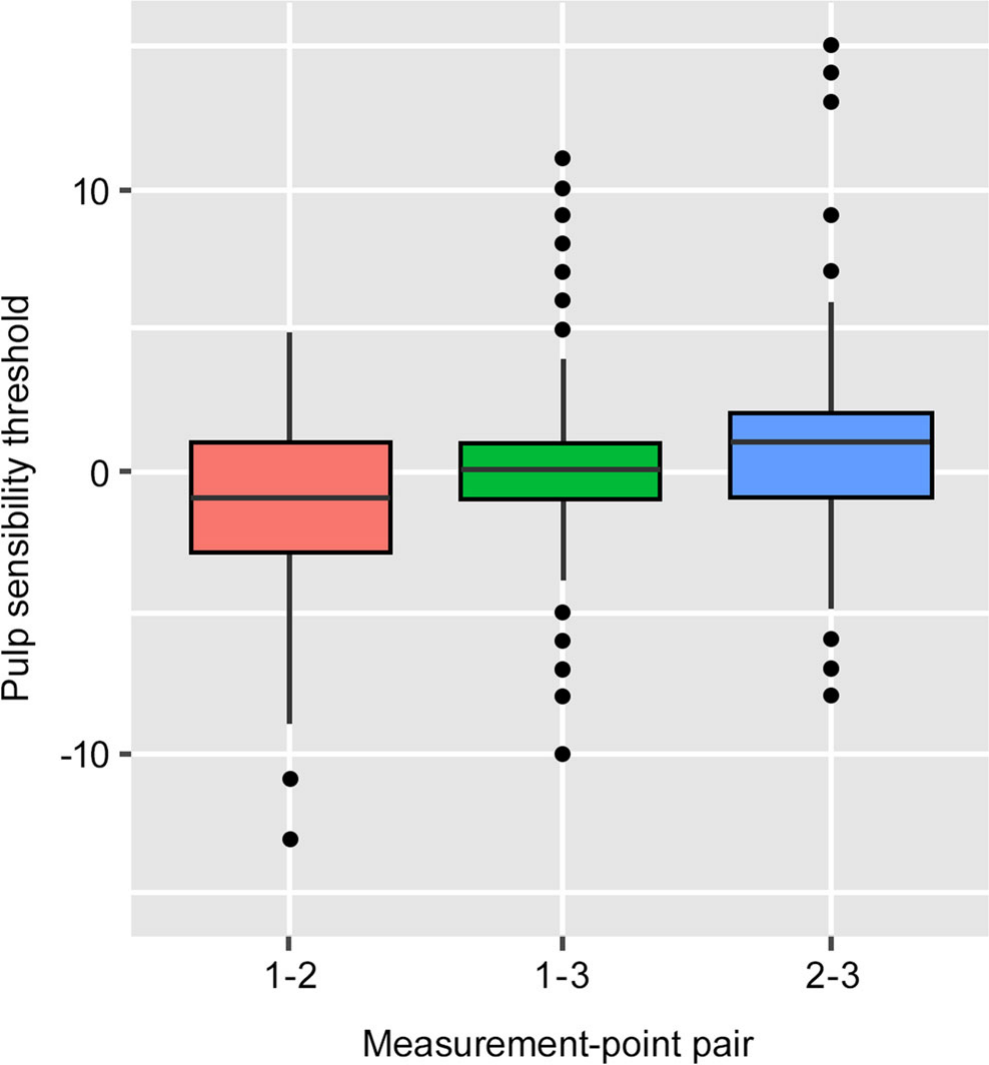
Box plot summarizing the distribution of the pulp sensibility threshold changes between the three different measurement-point pairs (1–2, 1–3, and 2–3). All three box plots are cover the zero-horizontal line, indicating that in a majority of patients there seems to be no considerable changes in the pulp sensibility thresholds between the three measurement-point pairs

No statistically significant differences were observed between the investigated groups. By means of a multilevel model output, the mean pulp sensibility threshold changes between the respective measurement points were equal to 0 (*p* values 1–2: 0.291; 1–3: 0.687; and 2–3: 0.146); thus, the null hypothesis could not be rejected (Table 2). Figures 2 and 3 depict box plots summarizing the sensibility threshold response differences of each measurement point and the three measurement-point pairs, respectively. The null hypothesis could not be rejected (Table 2). The fitted multilevel model found the mean pulp sensibility threshold changes between the respective measurement points not to be significantly different from 0 (*p* values 1–2: 0.291; 1–3: 0.687; and 2–3: 0.146). The box plots in Figs. 2 and 3 summarize the measured sensibility threshold responses at each measurement point as well as the changes of these values between the three measurement-point pairs, respectively.

**Table 2:** According to the null hypothesis the mean pulp sensibility threshold changes between the respective measurement-point pairs are equal to 0. Since all *p* values are larger than all usual significance levels, it can be concluded that there is insufficient evidence to show that the pulp sensibility threshold changes are significantly different from 0. (MPP = measurement-point pair, Est. = estimate, S.E. = standard error, *t* val. = *t* value, d.f. = degrees of freedom, *p* = p value, Std. Dev. = standard deviation)

	Fixed effects				
	Est.	S.E	*t* val.	d.f.	*p*
MPP 1-2	− 0.614	0.576	− 1.066	63000	0.291
MPP 1-3	0.233	0.576	0.405	63000	0.687
MPP 2-3	0.847	0.576	1.417	63000	0.146
	Random effects				
	Parameter	Std. Dev.			
MPP	(Intercept)	2.288			
Residual		3.565

## Discussion

The influence that orthodontic forces can have on cementum hardness and elastic modulus [14], root resorption induction, or pulp tissue changes [17–22, 25, 28, 30, 31] has been reported in the literature. Based on the scientific evidence and clinical perspectives, one can speculate that the dental pulp vitality during orthodontic treatment could be compromised, depending on the degree and time duration of the applied orthodontic forces on the involved teeth. An investigative model, therefore, was designed with the expectation that if the application of orthodontic forces would produce pulp tissue changes, they could be perceived by means of an electric pulp tester. Complete innervation of the dental pulp should be completed 4 to 5 years after eruption [39], even if the apex appears radiologically completely formed [40]. Bender et al. [41] suggested that a pulp stimulus response threshold correlates with its neural density. It can therefore be expected that younger pulps will respond with a higher stimulus amount. The teeth included in this study were considered to have complete morphological development. As a result, development immaturity was not considered as an influence to the electric stimuli response threshold in this investigation. The results’ consistency in this investigation—prior to the application of any orthodontic forces and during the investigation—supports this assumption.

In this investigation, compared to similar ones [20, 42, 43], pulp sensitivity was not tested by means of thermal tests since the comparison between the results obtained at the different measuring times would be subjective. Moreover, from a clinical point of view, all conventional pulp testing methods, specially thermal tests, not always return a yes/no but, frequently a dubious “maybe” response. Therefore, the inclusion of thermal tests as further pulp status diagnosis parameter in this study would not have been compatible with the aims of the study, due to their missing results quantification. Thus, this investigation was designed to allow a quantifiable comparison, to a certain degree, of an electric pulp tester stimuli threshold response on the same tooth at different measuring times and under different clinical conditions caused by applied orthodontic forces. Hence, a comparison between the results obtained with an electric pulp tester and the thermal results was not quantifiable. Furthermore, since the cold test false-negative and false-positive responses were not distinguishable [20, 42, 43], an uncontrollable variable would have been introduced. The use of a control group would likewise have been problematic when analyzing the results, since, in accordance with the inclusion protocol, it was expected that all teeth included in this investigation were clinically healthy and no orthodontic forces could have been applied to the control group. The inclusion of a control group with individuals not undergoing orthodontic treatment would have only allowed a yes/no stimuli response comparison between this group and the research group at baseline. The second and third measurements in a control group would have been also a subjective yes/no response, thus, being these results not objectively comparable with either the baseline ones of the control group or the ones of the second and third measurements of the research group.

The electric pulp tester employed in this investigation is clinically readily available, possesses an integrated, automatic stimulus intensity increase unit scale, and is easy to operate. Electric pulp tester literature communications are contrasting; it has been reported that the threshold sensation it produces is not painful [44], that it is an efficient and trustable pulp sensitivity testing method [45–48] with a similar reliability as cold pulp testing [42], and that the subjective pain ratings and the increment of pulp sensitivity measured with an electric pulp tester can be correlated [49]. Nevertheless, significant differences between the electric and cold tests by means of the VAS scale have been reported [50]. It has also been suggested that electric pulp testing is of little value when attempting to quantify nervous perception over repeated trials [47] and that electric pulp test results should be cautiously evaluated due to the possibility of incorrect pulp necrosis assumptions caused by incorrect interpretations of false-negative results [42, 49]. We are also of the opinion that such contrasting results [42, 47, 50] may be attributed to the different pulp reactions as well as different individual pain perceptions against different stimuli types. Based on the results obtained in this investigation and alike a previous report [47] we also recommend practitioners to be always aware that different electric pulp tester unit readings at different measuring times do not give any information concerning the histopathological status of the pulp, and that even if a clear negative reading is obtained, the electric pulp test per se is never recommended to be implemented as a conclusive diagnosis. According to the manufacturer [51], the normal stimulus unit response threshold ranges for incisors is between 10 and 40, 20 and 50 for bicuspids, and 30 to 70 for molars. The results obtained in this research are, depending on the tolerance allowed, relatively close to the ones given by the manufacturers. One notable exemption is the molars for which we never obtained a reading of more than 56 units. The manufacturer also states that the voltage of the electric pulp tester is electronically stabilized. It therefore does not allow the battery intrinsically to influence the electric pulp tester’s performance. The batteries of the device employed in this investigation were nonetheless renewed on each investigation day in order to avoid possible research parameter influences. Furthermore, the tooth site application of the electric pulp tester probe tip was determined prior to the baseline measurement in order to reproduce the measurements at all times [38, 52, 53]. Care was also taken to avoid any contact between the probe tip and/or conductive medium and the orthodontic attachments or neighbor hard or soft tissues. In this investigation, the stimulus increase rate of the electric pulp tester was set at 3, at which the peak voltage should reach 35 s after providing a constant stimulus intensity increase, thus enhancing the reproducibility and accuracy of the measurements [54].

From a logical, clinical stand point—and without taking into consideration all of the possible inherent subjective factors—if the electric stimulus response is delayed or increased that would clinically mean that the pulp tissues are inflamed, since it takes longer (higher device unit value) to obtain a response to the electric stimulus. On the contrary, the sooner a pulp would respond to an electrical stimulus (lower device unit value) the “healthier” it should be. In this study, a decreased response threshold means a fast response (low device unit value) to an electrical stimulus. It must be stressed, however, that this is only an assumption in an effort to correlate our results with the ones given in the literature and it should not be associated with the histopathological status of a pulp [47]. Thus, the quantifiable differences obtained with the electric pulp tester between baseline and second and/or third measurements in this investigation should not be interpreted as a pulp health status difference but rather as the possibility that physiological changes in the pulp, caused by the application of orthodontic forces, could be taking place. It is a common belief that a missing response to an electrical stimulus indicates a non-vital pulp. However, results reported by various authors [41, 43, 52] indicate that there is a period of time after orthodontic movement or trauma in which an electric pulp stimulus response threshold may be so increased that no response is possible. On the other hand, Fulling and Andreasen [45] report that the response threshold to an electric pulp tester did not change or that no stimulus response was obtained at all, depending on the electric pulp tester employed. In this investigation—contrary to other reports [20, 42] and in accordance to others [43, 52, 55] with a similar investigation aim—all teeth responded to the electric pulp tester at all times. This, in turn, allowed for the use of the response thresholds obtained at the baseline as comparison values with the results after orthodontic forces were applied.

The mean response thresholds—measured immediately after ligation of the initial nickel titanium archwire—increased in all teeth groups. These results are in accordance with the results obtained by different authors [41, 43, 52, 55]. Cave et al. [42] also reported an increased stimuli response threshold to an electric pulp tester shortly after the orthodontic force was applied. But contrary to our observations, they also reported that it remained at high levels up to 9 months afterward and that they observed negative responses to electric stimulus. They concluded, similar to Han et al. [20], that since they always obtained a response with the cold test, it could be a more reliable one during orthodontic treatment. However, although a yes/no stimuli response might suggest, from a clinical point of view, a straightforward interpretation, that from a scientific point of view is an unreliable research parameter since it is not quantifiable. From our investigation one can conclude—even if statistically non-significant results were obtained between the baseline and further measurements, and only tendencies are discernible—that such differences suggest alterations occurred within the pulp tissue. Such a response threshold increment could be explained as the result of pressure or tension applied on the pulp apical nerve fibers [43, 52] and to a certain extent, because post-traumatic teeth do not always respond immediately to an electric stimulus [41]. However, we believe that such observations do not indicate a loss of pulpal vitality [47], at least in healthy, non-traumatized pulps.

In this study, we observed a decreasing electric stimuli response threshold tendency after 9 to 15 months of using the nickel titanium and placing the stainless steel archwires. Similar observations were reported by different researchers [42, 55], using a similar investigation method when evaluating electric pulp test responses during orthodontic treatment. Statistically significant differences between mean electrical thresholds following orthodontic activation also were not observed in other investigations [9, 49] with similar methodology. On the other hand, other researchers report significant differences at various electric pulp testing stages [20, 52]. That said, all of these electric stimulus studies suggest a tendency for pulp recovery over time during orthodontic treatment. In accordance with the observations reported by other investigators [18, 36, 56], possible pulp sensitivity changes were measured almost immediately after an orthodontic load was applied. An electric pulp tester unit increment response threshold tendency was observed after the nickel titanium was applied. It would have been interesting to assess if an electric stimulus increment response took place after the ligature of the stainless steel archwire. However, this measurement was not possible since the treatment protocol was designed on a case by case basis. The manipulation, as well as the time elapsed between the nickel titanium and stainless steel archwires, was thus different so that an uncontrollable variable would have been introduced.

In this investigation, a consistent stimuli response threshold decreasing tendency was observed in all teeth groups. The maxillary first molar [16] appeared to have the slowest, while the mandibular first incisor [31] and canine [33] showed the fastest electric stimuli recovery tendencies. These results are similar to those in anterior teeth reported by Burnside et al. [52], yet they report a statistically significant response threshold. The continued response threshold decrease observed in the maxillary and mandibular incisors in this study could be caused, when compared with premolars and molars, as a result of their smaller crown and root surface area, thus bearing a higher pressure in the apical area [43, 52]. Furthermore, these tendencies could be explained through the different individual orthodontic force amount required and morphological conditions [41]. It should also be taken into consideration that individual tooth pain response threshold also depends on the individual subjectivity—a variable that was not taken into consideration in this investigation. Similar observations have been reported by Cave et al. [42].

An ideal mean force for tipping of canines and premolars, respectively, of 62.5 cN (30 to100 cN) and 56.1 cN (range 30 to 100 cN) [57] was recommended, depending on the particular root surface area. Excessive orthodontic forces were not employed in this investigation, which could account for why no statistical significances and only tendencies could be observed. While no statistically significant results could be calculated in this investigation—and contrary to the results reported by other studies [20, 42, 52, 55],different stimuli response threshold tendencies could be observed. The agreement between this research and other studies with similar designs [20, 22, 42, 43, 49, 52, 55] is only partial. These differences could be explained via methodological differences, results interpretation, and the employment of different electric pulp testers [58]. The different electric stimulus response thresholds observed in this investigation between the different teeth types could be explained through the nerve number, size and orientation, possible temperature and humidity changes, enamel and/or dentin thickness, and patient subjectiveness. Another rationale to explain these differences could be the different archwire types and thickness used in this study (0.014” or 0.016”). It has nevertheless been reported that pain caused during an orthodontic treatment is subjective and is not related to the dental archwire [59]. It could therefore be assumed that the archwire type and diameter difference did not influence the electric stimulus threshold in this study.

An objective, quantifiable clinical evaluation of pulp tissue inflammatory alterations may not be feasible today due to the complex factors associated with the morphology and physiology of dental pulp. However, the results of this investigation suggest the possibility to detect on time an excessive orthodontic force application when routinely screening pulp status during orthodontic treatment, by means of an electric pulp tester.

## Conclusions

1. The results obtained in this investigation revealed differences of electric stimulus response threshold of the pulp caused during orthodontic treatment, between measurements made at baseline and immediately after the placement of nickel titanium and stainless steel archwires.
2. Pulpal electric stimulus response threshold is influenced by the application of orthodontic forces.
3. All teeth types investigated showed a statistically non-significant mean response threshold decrease when comparing the third with the baseline measurement.
4. A decreasing threshold was observed in all teeth after the second measurement, and a threshold increase in the third measurement.
5. The slowest electric stimuli mean response threshold recovery was observed in tooth 16 and the fastest in teeth 31 and 33.

## References

[1] Dummer PM, Hicks R, Huws D (1980). Clinical signs and symptoms in pulp disease. Int Endod J.

[2] Mumford JM (1967). Pain perception threshold on stimulating human teeth and the histological condition of the pulp. Br Dent J.

[3] Seltzer S, Bender IB, Ziontz M (1963). The dynamics of pulp inflammation: correlations between diagnostic data and actual histologic findings in the pulp. Oral Surg Oral Med Oral Pathol.

[4] Magitot E (1878). Treatise on dental caries.

[5] Marshall JS (1891). Electricity as a therapeutic agent in the hyperemia and congestion of the pulp and peridental membrane. Dent Cosmos.

[6] Prinz H (1919). Diseases of the dental pulp. I. Diagnosis. Dent Cosmos.

[7] Ziskin DE, Wald A (1938). Observations on electrical pulp testing. J Dent Res.

[8] Kaletsky T, Furedi A (1935). Reliability of various types of pulp testers as a diagnostic aid. J Am Dent Assoc.

[9] Markus MB (1946). The reaction of the pulp to pressure. Am J Orthod.

[10] Lundy T, Stanley HR (1969). Correlation of pulpal histopathology and clinical symptoms in human teeth subjected to experimental irritation. Oral Surg Oral Med Oral Pathol.

[11] Bjorn H (1946). Electrical excitation of the tooth and its application in dentistry. Sven Tandlak Tidskr.

[12] Dummer PM, Tanner M (1986). The response of caries-free, unfilled teeth to electrical excitation: a comparison of two new pulp testers. Int Endod J.

[13] Rowe AH, Pitt Ford TR (1990). The assessment of pulpal vitality. Int Endod J.

[14] Srivicharnkul P, Kharbanda OP, Swain MV, Petocz P, Derendeliler MA (2005). Physical properties of root cementum: Part 3. Hardness and elastic modulus after application of light and heavy forces. Am J Orthod Dentofac Orthop.

[15] Romanyk DL, Melenka GW, Carey JP (2013). Modeling stress-relaxation behavior of the periodontal ligament during the initial phase of orthodontic treatment. J Biomech Eng.

[16] Yamaguchi M, Kasai K (2005). Inflammation in periodontal tissues in response to mechanical forces. Arch Immunol Ther Exp (Warsz).

[17] Hamersky PA, Weimer AD, Taintor JF (1980). The effect of orthodontic force application on the pulpal tissue respiration rate in the human premolar. Am J Orthod.

[18] McDonald F, Pitt Ford TR (1994). Blood flow changes in permanent maxillary canines during retraction. Eur J Orthod.

[19] Mengel MK, Stiefenhofer AE, Jyväsjärvi E, Kniffki KD (1993). Pain sensation during cold stimulation of the teeth: differential reflection of A delta and C fibre activity?. Pain.

[20] Han G, Hu M, Zhang Y, Jiang H (2013). Pulp vitality and histologic changes in human dental pulp after the application of moderate and severe intrusive orthodontic forces. Am J Orthod Dentofac Orthop.

[21] Perinetti G, Varvara G, Festa F, Esposito P (2004). Aspartate aminotransferase activity in pulp of orthodontically treated teeth. Am J Orthod Dentofac Orthop.

[22] Veberiene R, Smailiene D, Danielyte J, Tolekis A, Dagys A, Machiulskiene V (2009). Effects of intrusive force on selected determinants of pulp vitality. Angle Orthod.

[23] Sano Y, Ikawa M, Sugawara J, Horiuchi H, Mitani H (2002). The effect of continuous intrusive force on human pulpal blood flow. Eur J Orthod.

[24] Ramazanzadeh BA, Sahhafian AA, Mohtasham N, Hassanzadeh N, Jahanbin A, Shaleri MT (2009). Histological changes in human dental pulp following application of intrusive and extrusive orthodontic forces. J Oral Sci.

[25] Caviedes-Bucheli J, Moreno JO, Ardila-Pinto J, Del Toro-Carreño HR, Saltarín-Quintero H, Sierra-Tapias CL, Macias-Gomez F, Ulate E, Lombana-Sanchez N, Muñoz HR (2011). The effect of orthodontic forces on calcitonin gene-related peptide expression in human dental pulp. J Endod.

[26] Derringer KA, Linden RWA (2004). Vascular endothelial growth factor, fibroblast growth factor 2, platelet derived growth factor and transforming growth factor beta released in human dental pulp following orthodontic force. Arch Oral Biol.

[27] Derringer KA, Linden RWA (2003). Angiogenic growth factors released in human dental pulp following orthodontic force. Arch Oral Biol.

[28] Derringer KA, Jaggers DC, Linden RW (1996). Angiogenesis in human dental pulp following orthodontic tooth movement. J Dent Res.

[29] Derringer K, Linden R (2007). Epidermal growth factor released in human dental pulp following orthodontic force. Eur J Orthod.

[30] Kvinnsland I, Kvinnsland S (1990). Changes in CGRP-immunoreactive nerve fibres during experimental tooth movement in rats. Eur J Orthod.

[31] Parris WG, Tanzer FS, Fridland GH, Harris EF, Killmar J, Desideiro DM (1989). Effects of orthodontic force on methionine enkephalin and substance P concentrations in human pulpal tissue. Am J Orthod Dentofac Orthop.

[32] Başaran G, Ozer T, Kaya FA (2006). Interleukine-1beta and tumor necrosis factor-alpha levels in the human gingival sulcus during orthodontic treatment. Angle Orthod.

[33] van Gastel J, Teughels W, Quirynen M, Struyf S, Van Dame J, Coucke W, Carine C (2011). Longitudinal changes in gingival crevicular fluid after placement of fixed orthodontic appliances. Am J Orthod Dentofac Orthop.

[34] Ren Y, Hazemeijer H, de Haan B, Qu N, De Vos P (2007). Cytokine profiles in crevicular fluid during orthodontic tooth movement of short and long durations. J Periodontol.

[35] Barwick PJ, Ramsay DS (1996). Effect of brief intrusive force on human pulpal blood flow. Am J Orthod Dentofac Orthop.

[36] Brodin P, Linge L, Aars H (1996). Instant assessment of pulpal blood flow after orthodontic force application. J Orofac Orthop.

[37] Consolaro A, Bianco Consolaro R (2018). There is no pulp necrosis or calcific metamorphosis of pulp induced by orthodontic treatment: biological basis. Dental Press J Orthod.

[38] Lin J, Chandler N, Purton D, Monteith B (2007). Appropriate electrode placement site for electric pulp testing first molar teeth. J Endod.

[39] Johnsen DC (1985) Innervation of teeth: Qualitative, quantitative, and developmental assessment. J Dent Res 64 Spec No:555–563. doi: 10.1177/002203458506400410 10.1177/002203458506400410 3857257

[40] Klein H (1978). Pulp responses to an electric pulp stimulator in the developing permanent anterior dentition. ASDC J Dent Child.

[41] Bender IB, Landau MA, Fonsecca S, Trowbridge HO (1989). The optimum placement-site of the electrode in electric pulp testing of the 12 anterior teeth. J Am Dent Assoc.

[42] Cave SG, Freer TJ, Podlich HM (2002). Pulp-test responses in orthodontic patients. Aust Orthod J.

[43] Hall CJ, Freer TJ (1998). The effects of early orthodontic force application on pulp test responses. Aust Dent J.

[44] Kleier DJ, Sexton JR, Averbach RE (1982). Electronic and clinical comparison of pulp testers. J Dent Res.

[45] Fulling HJ, Andreasen JO (1976). Influence of maturation status and tooth type of permanent teeth upon electrometric and thermal pulp testing. Scand J Dent Res.

[46] Fuss Z, Trowbridge H, Bender IB, Rickoff B, Sorin S (1986). Assessment of reliability of electrical and thermal pulp testing agents. J Endod.

[47] Lado EA, Richmond AF, Marks RG (1988). Reliability and validity of a digital pulp tester as a test standard for measuring sensory perception. J Endod.

[48] Moore PA, Boynes SG, Hersh EV, DeRossi SS, Sollecito T, Goodson JM, Leonel J, Floros C, Peterson C, Hutcheson M (2006). The anesthetic efficacy of 4 percent articaine 1:200,000 epinephrine: two controlled clinical trials. J Am Dent Assoc.

[49] Leavitt AH, King GJ, Ramsay DS, Jackson DL (2002). A longitudinal evaluation of pulpal pain during orthodontic tooth movement. Orthod Craniofacial Res.

[50] Dalili M, Närhi M, Laine-Alava R, Myllykangasn R (1998). Dental electrical thresholds and cold sensitivity during orthodontic therapy. J Dent Res.

[51] SybronEndo Vitality Scanner Model (2006) Instruction guidelines. SybronEndo, Glendora, CA, USA. https://embed.widencdn.net/download/kavokerr/v1ti9tnbcp/VITALITY-SCANNER-IFU-300-520-REV-G-WEB.pdf?u=18sth1

[52] Burnside RR, Sorenson FM, Buck DL (1974). Electric vitality testing in orthodontic patients. Angle Orthod.

[53] Mickel AK, Lindquist KAD, Chogle S, Jones JJ, Curd F (2006). Electric pulp tester conductance through various interface media. J Endod.

[54] Abdel Wahab MH, Kennedy JG (1987). The effect of rate of increase of electrical current on the sensation thresholds of teeth. J Dent Res.

[55] Alomari FA, Al-Habahbeh R, Alsakarna BK (2011). Responses of pulp sensibility tests during orthodontic treatment and retention. Int Endod J.

[56] Ikawa M, Fujiwara M, Horiuchi H, Shimauchi H (2001). The effect of short-term tooth intrusion on human pulpal blood flow measured by laser Doppler flowmetry. Arch Oral Biol.

[57] Kurol J, Franke P, Lundgren D, Owman-Moll P (1996). Force magnitude applied by orthodontists. An inter- and intra-individual study. Eur J Orthod.

[58] Brown AG, Kloka AC, Beeler WJ (1984). Sensory response to constant electrical stimulation of teeth. J Dent Res.

[59] Jones M, Chan C (1992). The pain and discomfort experienced during orthodontic treatment: a randomized controlled clinical trial of two initial aligning arch wires. Am J Orthod Dentofac Orthop.

